# Clinician-administrator; challenges associated with changing roles

**DOI:** 10.15694/mep.2017.000099

**Published:** 2017-06-13

**Authors:** Sarath Lekamwasam

**Affiliations:** 1 Faculty of Medicine

**Keywords:** clinician-administrator, dean, medical education

## Abstract

This article was migrated. The article was marked as recommended.

Abstract

Rapid changes in disease pattern, advancement of medical technology and introduction of new treatment modalities demand continuous modifications in medical education. This can be a daunting task unless all stakeholders engaged in medical education are convinced that such changes are necessary to ensure the efficiency of the end product. The task of leading a group of academics who themselves are leaders and have their personal targets in the direction of a common goal can be a challenge. This, however, becomes the primary responsibility of a Medical Dean. In order to achieve this, Dean has to have leadership qualities and particularly good communication skills. Whether these factors are taken into consideration in selecting a Dean is questionable. Further, no attempts are made to enhance leadership qualities and other attributes that a Dean should possess among potential candidates of deanship.

## Clinician-administrator; challenges associated with changing roles

### Background

Medical education has undergone notable secular changes in response to a variety of factors including changing disease pattern, medical inventions and new therapeutic interventions. Despite these, there is a global concern that medical education and training of health professionals are not keeping pace with the health and other challenges in the present day. Special efforts are being made to reorganize medical education to improve the quality, relevance and social accountability ( Pershing S, Fuche VR 2013). These modifications in medical undergraduate training require a strong commitment and support of all categories involved in undergraduate education and more importantly, Dean as the Head of the institution. Hence the role of Dean in a medical faculty needs a closer review.

### Discussion

Dean who holds the highest administrative position is selected from the department Heads for a period of three years and the office is renewable. As no criteria or guidelines exist in selecting a Dean, the appointment is made after consensus or vote among academia. There is no mechanism to check whether the dean-to-be possesses correct attributes and qualities that are required to lead the institution. Dean has to lead a group of academics who themselves are leaders in their respective study fields, and keep them together focused on the strategic plan and goals of the institution. Leading a group of academics who have different aspirations and requirements in one pathway towards a common goal can be a daunting task.

Although Dean has to remain focused on a common goal, university academics are self-centered and are more interested in personal goals and targets. Their promotions are based on individual performances and achievements. Securing grants also depends on individual competencies and achievements. Hence an academic who was pursuing personal goals has to swiftly shift the pattern of thinking and priorities once selected to the post of Dean. Situation can be more complex when a clinician is selected to the post of medical Dean. Clinicians make crucial decisions on complex clinical situations, on critically ill patients and on matters that influence the death or survival of patients. These management decisions in clinical medicine are mostly made individually and with a certain degree of autonomy. Although multidisciplinary team approach in patient care is widely practiced in many other countries, in Sri Lanka mostly clinicians make clinical decisions individually. Furthermore, clinician occupies a hierarchically high position in the management team and is the major decision maker in a given situation. Therefore clinician may find it more difficult to change his role from clinician to administrator.

Although there are no guidelines for the selection process, Dean in a medical faculty is expected to possess certain qualities. Eugene *et al* in 2008, published the desirable qualities of a medical school Dean ( Eugene, *et al* 2008). In this, management skills and leadership qualities are identified as the most desirable qualities and given the responsibilities and complexity of day to day work of a medical Dean this can be easily understood.

Opportunities for clinicians to enhance their managerial skills are scarce. Medical undergraduate curriculum does not address this area possibly due to the fact that these extra skills are not essential attributes of a basic doctor. During the pathway of postgraduate training clinicians are trained to provide care for individual patients and no serious attempt is made to enhance managerial skills. Hence clinician needs to acquire managerial skills on-the-job, quickly, once selected to the post.

Under the management skills, communication skills also take a high priority ( Eugene, *et al* 2008). It is not surprising to give such prominence to communication skills as medical Dean has to make a good rapport with a diverse group of stakeholders. The bulk of the clinical training of medical undergraduates is done by clinicians attached to hospitals and an effective communication channel is an essential requirement for smoother running of the clinical training program. Additionally, effective communication methods are required when dealing with critical masses such as students and non-academics. Clinician-administrator has an advantage as communication skill is an attribute that every clinician is expected to acquire during the training and practice in day-to-day clinical work. Current specialist training places a great emphasis on the principles of communication and these skills are assessed in formative assessments in postgraduate examinations.

Medical education undergoes secular changes and it is the duty of Dean to make sure that necessary modifications are included in the curriculum in order to keep the curriculum attractive and acceptable to concerned parties. Curriculum needs to be revisited, updated and aligned to suit the objectives and mission and vision statements of the institution and more importantly country’s health demand. Making significant changes in the curriculum and obtaining the approval and support of all academics can be a difficult task.

In the field of administration, there are managers and leaders. An adage attributed to Warren G Bennis makes a clear distinction between the two categories. According to him “manager does things right while leader does right things”. Although Dean should preferably be a leader than a manger there are many challenges a medical Dean has to face when working in this capacity. During a semi-structured interview involving 18 medical Deans in the USA, 14(78%) identified financial difficulties as the most pressing leadership challenge. Other challenges identified were staffing problems (33%) and poor morale (28%) and these three would be the most common challenges that a local medical Dean also has to face ( Wiley W Souba, David V Day 2006).

Clinician needs to change or make adjustments in the decision making process that he has been familiar with once his duties are changed. Clinicians are trained to make decisions on individual patients within an acceptable time period. These decisions are made based on accepted set of principles. Although clinical experience was the major factor in clinical decision making in the past, concepts such as evidence-based medicine and cost-effectiveness play a major role in clinical decision making in this era. Evidence-based medicine is a well accepted concept in modern medicine and it has made inroads to other areas such as evidence-based nursing and evidence-based psychotherapy. Evidence-based decisions in healthcare are made after a thorough analysis of research data often in the form of meta-analyses. Evidence-based management strategies are not being widely used in the undergraduate education in Sri Lanka. There is a growing interest among many higher education institutes to modify their teaching and learning activities based on research evidence. The evidence based teachers network in the UK ( http://www.ebtn.org.uk) has recognized ten evidence-based teaching learning activities that would make a high impact on students. Evidence-based education where all aspects of education, from policy making to classroom practices, are based on research evidence is not widely practiced in many higher education institutes including Sri Lanka.

Clinician, however, possibly can impart certain skills developed over years to the administration. One such skill is the methods of risk minimization used in health care systems. Accidents and errors in health care are not new and certainly not uncommon. Many checks and balances are kept in place and a great caution is exercised by all concerned parties to ensure patient safety. The Swiss cheese model introduced by James Reason in 1990 ( Reason J 1990) highlights how the system fails and the error escapes all barriers kept in place at a given moment. Here, slices represent the barriers or defenses against errors and holes in slices represent their inherent weaknesses. These weaknesses vary in size and position not allowing an error to go through all defenses. However when all holes in each slice momentarily align, error can finds it trajectory (figure). Apart from clinical medicine, the Swiss cheese model is applied in many fields such as aviation industry and engineering and there no reason why it cannot be applied to university administration. It is logical to identify high-risk areas related to the administration in higher education institutes and ensure that adequate barriers are placed to prevent accidents and errors. Matters related to evaluations, examination results, finances and handling of hazardous materials are some such high risk areas.

Furthermore, various appraisal methods have been introduced for clinicians particularly for those in the training and in some countries they are mandatory. These methods assess the performance of clinicians in quantitative and qualitative manner. Although the competence of clinicians can be assessed by analyzing the outcome in quantitative manner it is equally important to assess the behavioral competence. The 360-Degree performance assessment or multisource feedback (MSF) which is entirely based on input from peers is often used to assess the behavioral competence of clinicians. It collects feedbacks from multiple sources on selected domains of performances and compares with the self-assessment. It is important to note that the same method is being used as a method of formative assessment of Deans of higher education institutes in certain countries. They are done at specified times often in cyclical manner. The 360-Degree assessment is easy to perform and its validity and reliability in assessing doctors have been established ( Paul Lelliott, et al 2008). It is important to consider cultural adaption of this assessment method before implementing. Furthermore, the suitability of adopting the 180-Degree of performance assessment which has input both from peers and superiors needs to be considered.

### Summary

Effective communication skills, leadership qualities, flexibility, patience and ability to listen to others are the basic qualities that a medical Dean should possess. First class temperament would probably be a more suitable qualification than a first class degree for a Dean. More opportunities should be allowed for deans-to-be to develop leadership qualities and sharpen their administrative qualities. Although there are no guidelines to select a medical Dean, the 14 items proposed by Dr Robert M Daugherty ( Robert M Daugherty Jr 2014) provide a reasonable checklist for prospective candidates to check their readiness to accept the office. Although it is easy to manage a medical faculty, leading a group of leaders (or herding cats) to a common goal could be a daunting task.

## Notes On Contributors

Author is the Chair Professor of Medicine with more than 20ys of clinical experience. Served as the Dean of the Faculty of Medcine, Univeristy of Ruhuna for three years. Has a keen interest in methods of clinical decision making and evidence based medicine.
