# Supplementary material for: Clinician-administrator; challenges associated with changing roles
Source: MedEdPublish (2016). 2017 Jun 13;6:99. [Version 1] doi: 10.15694/mep.2017.000099 (PMC10885261; doi:10.15694/mep.2017.000099)
Supplement: Peer review history

# Reviewer report: Reviewer response for version 1

**DOI:** 10.21956/mep.19261.r27985

**Published:** 2019-03-07

**Authors:** Trevor Gibbs

**Affiliations:** 1 AMEE

This review has been migrated. The reviewer awarded 4 stars out of 5 A very interesting and well written paper that covers a not-often discussed but important subject. I would agree with my co-author regarding the importance of country and context and the very different approaches to leadership. It seems that a vertical , hierarchical structure still exists in many regions of the world, where such a horizontal approach such as described may not be appreciated. I would be happy to recommend this paper to faculty moving into leadership positions and to those involved in leadership selection procedures.

Reviewer Expertise:

NA

No decision status is available

# Reviewer report: Reviewer response for version 1

**DOI:** 10.21956/mep.19261.r27986

**Published:** 2018-06-18

**Authors:** Anthony David.M

**Affiliations:** 1 Soho

This review has been migrated. The reviewer awarded 4 stars out of 5 A very useful article. Thank you for submitting it. Indeed the paucity of leadership training to medical students is lamentable. Some changes are being seen in some areas where Leadership is seen as one of the five key roles for a doctor. Yet modules clearly designed to impart education in terms of leadership qualities are on the anvil. Communication skills are very essential both for leaders of institutions as well as for team leaders, at a lower level. In fact all doctors need effective communication skills to maintain good relationships with patients and the public.

Reviewer Expertise:

NA

No decision status is available

# Reviewer report: Reviewer response for version 1

**DOI:** 10.21956/mep.19261.r27984

**Published:** 2017-06-20

**Authors:** Trudie Roberts

**Affiliations:** 1 Leeds Institute of Medical Education

This review has been migrated. The reviewer awarded 4 stars out of 5 This is an interesting and reflective piece from someone with considerable experience in a leadership post. I wonder further about leadership qualities being subject to context and culture. By this I mean are some qualities of a leader more valued in some cultures than others. For example the strong autocratic leader vs the collaborative, consensus leader? It would be interesting to develop this further. Perhaps it has already been done - I'm not an expert in this area.Re changes in medical education. I agree totally that we need to be more agile with designing the education students receive. The revolutionary change in healthcare delivery demands this. It can be difficult to push back against the self-interests of high profile individuals but involving stakeholders from outside the medical school such as patients, carers and health system designers is very useful. At the end of the day there are likely to be two sets of skills all doctors require.1. the here and now skills to equip students to cope with the health system they will enter2. the more important longer terms skills of critical thinking, collaboration, problem solving, resilience etc.

Reviewer Expertise:

NA

No decision status is available
